# An experimental game to assess hunter’s participation in zoonotic diseases surveillance

**DOI:** 10.1186/s12889-024-17696-7

**Published:** 2024-02-01

**Authors:** Aude Pouliquen, Gilles Aurélien Boupana Mapeyi, Hadrien Vanthomme, Marie-Marie Olive, Gaël Darren Maganga, Daniel Cornelis, Sébastien Lebel, Marisa Peyre, Alexis Delabouglise

**Affiliations:** 1 CIRAD, UMR ASTRE, Montpellier, France; 2 CIRAD, UMR ASTRE, Univ Montpellier, INRAE, Montpellier, France; 3 CIRAD, UPR Forêts Et Sociétés, Montpellier, France; 4 CIRAD, Forêts et Sociétés, Université de Montpellier, Montpellier, France; 5 Centre Interdisciplinaire de Recherches Médicales de Franceville (CIRMF), Franceville, Gabon

**Keywords:** Zoonotic disease surveillance, Wild meat supply chain, Experimental game, Participatory epidemiology, Gabon, Hunting, Wildlife health events

## Abstract

**Background:**

Strengthening the surveillance of zoonotic diseases emergence in the wild meat value chains is a critical component of the prevention of future health crises. Community hunters could act as first-line observers in zoonotic pathogens surveillance systems in wildlife, by reporting early signs of the possible presence of a disease in the game animals they observe and manipulate on a regular basis.

**Methods:**

An experimental game was developed and implemented in a forested area of Gabon, in central Africa. Our objective was to improve our understanding of community hunters' decision-making when finding signs of zoonotic diseases in game animals: would they report or dissimulate these findings to a health agency? 88 hunters, divided into 9 groups of 5 to 13 participants, participated in the game, which was run over 21 rounds. In each round the players participated in a simulated hunting trip during which they had a chance of capturing a wild animal displaying clinical signs of a zoonotic disease. When signs were visible, players had to decide whether to sell/consume the animal or to report it. The last option implied a lowered revenue from the hunt but an increased probability of early detection of zoonotic diseases with benefits for the entire group of hunters.

**Results:**

The results showed that false alerts—i.e. a suspect case not caused by a zoonotic disease—led to a decrease in the number of reports in the next round (Odds Ratio [OR]: 0.46, 95% Confidence Interval [CI]: 0.36–0.8, *p* < 0.01). Hunters who had an agricultural activity in addition to hunting reported suspect cases more often than others (OR: 2.05, 95% CI: 1.09–3.88, *p* < 0.03). The number of suspect case reports increased with the rank of the game round (Incremental OR: 1.11, CI: 1.06–1.17, *p* < 0.01) suggesting an increase in participants’ inclination to report throughout the game.

**Conclusion:**

Using experimental games presents an added value for improving the understanding of people’s decisions to participate in health surveillance systems.

**Supplementary Information:**

The online version contains supplementary material available at 10.1186/s12889-024-17696-7.

## Background

Zoonotic diseases have accounted for 60% of emerging disease events since the middle of the twentieth century [1, 2]. The majority of these events have an origin in wildlife [1, 3, 4] as exemplified by the epidemics of Ebola virus disease [5, 6] and Nipah virus [7]. More recently wildlife has been highly suspected of being the original reservoir of the SARS-CoV-2 virus [8–11].

Human-wildlife interactions are facilitated by wild animal hunting and trade, processing and consumption of wildlife products, a widespread practice in Sub-Saharan Africa [12–14]. Wild meat is the primary source of protein for hunters and their families and represents the unique source of income of a large fraction of households [12, 13, 15, 16]. The close contact of communities with live wild animals and their wildlife products make them particularly vulnerable to zoonotic disease transmission [17, 18]. Notably, in some of the past outbreaks of Ebola virus, the initial viral transmission from wildlife to humans was mediated by the hunting and handling of infected wild animals [19–21]. Hunters communities are also first-line observers of early signs of disease infection in wild animals and human cases of zoonotic diseases transmitted from wildlife [22]. To adequately manage the risk of transmission of such diseases, reliable warning systems reporting early signs of the presence of zoonoses are needed [10, 23]. In this regard, community-based surveillance systems have attracted a lot of interest [17, 22, 24–28]. These systems rely on the clinical observations made by local communities and their knowledge of diseases affecting animal populations for the early detection of pathogens’ spillovers towards human population and for engaging rapid control measures [22, 26, 29]. A community-based zoonosis surveillance system relies on the observation of a set of symptoms, in animals or in humans, that correspond to the definition of a suspect case of zoonosis.

Several studies showed that the risk of disease transmission from wild animals to humans is unequally perceived by local communities [25, 26]. It was evaluated in surveys conducted in Sub-Saharan African countries targeting hunters, butchers, sellers and consumers [30–32], with significantly different results: 24% of the respondents were aware of zoonotic risks in a survey conducted in Sierra Leone [32]; 55% in Nigeria [33]; and 74% in southern Cameroon [31]. While a limited knowledge may constitute a first barrier to the establishment of a community-based surveillance system, it can be improved through awareness campaigns and education programs [25, 32]. However, participation in health surveillance is also affected by a range of other factors, including the anticipated direct or indirect costs of information disclosure to the health agency and the value attributed to the intervention of the health agencies [34–36]. To the authors’ knowledge, peoples’ attitude towards wildlife health surveillance was not thoroughly investigated. One study by Guenin et al*.* [26] conducted in Guinea identified possible communication channels and clinical observations relevant to local communities living in proximity to wildlife. However, the willingness of wild meat value chain actors to participate in zoonoses surveillance has not been formally assessed yet. In the context of animal husbandry, farmers anticipate negative consequences associated with disease reporting that include the prohibition of selling animals, reduction in market prices due to disease announcement, mandatory administrative slaughter of valuable animals, and administrative procedures, while benefits include the reduction of health risks for the community [35–37]. From the hunters’ perspective, reporting a suspected disease observed on a game animal implies foregoing the benefits of using the animal for their own consumption or for sale [26]. On the other hand, if a zoonosis is present, early detection allowed by reporting will benefit the whole community by reducing the chances of a community member being infected. The benefits include the avoidance of diseases with severe welfare consequences and the saved costs of hospitalisation, medication and law restriction associated to hunting activity. Consequently, if hunters are convinced that the information they provide will produce benefits for disease control interventions, their participation in zoonoses surveillance involves a social dilemma between the provision of food or income to their household and the health protection of the community.

Experimental games (EGs) provide information about the decision-making process of a population of players faced with hypothetical scenarios and the necessity to choose among different options. Frequently, these decisions involve the management of a common or public goods and the player’s choices favour either their own or the community’s interests [38–42]. The observations of players’ behaviour under experimental conditions are compared to predictions of game-theory models that assume players are rational utility-maximisers [43]. EGs were used to study the adoption of health improving behaviours in a context of strategic interactions, i.e. when the choices made by some players affect the health risk exposure of other players and, in turn, the anticipated benefits or costs associated with actions related to health. Several experiments on the adoption of vaccination against infectious human diseases showed the existence of a free-rider behaviour amongst players. Extrapolated to real-life situations, this behaviour may explain failures to reach a vaccination coverage allowing a complete disease control as the reduction in infection risk resulting from herd immunity results in a decreased willingness to vaccinate [44–47]. To the authors’ knowledge this methodological approach was not applied to study the participation in health surveillance systems.

An EG simulating the implementation of a community-based surveillance system of zoonotic diseases in wildlife based on the voluntary report of hunters was developed and tested. The game was designed to be played by groups of commercial or subsistence hunters. The objective was to identify the characteristics of hunters (their socioeconomic features) and of epidemiological and surveillance processes (occurrence of zoonotic diseases, true and false disease alerts and resulting losses for the hunters) that may affect their likelihood to participate in wildlife diseases surveillance. 

## Material and methods

### Study area and sampling strategy

The study was implemented in the department of Mulundu, located in the province of Ogooué-Lolo in Gabon. The study area is one where hunting, which is widespread, is not prohibited but regulated. Hunting, practiced year-round, is the primary source of income for most households and the main source of proteins. The most commonly hunted animals are duikers, porcupine, red- river hogs and monkeys. Considering the low human density of the intervention site, a sustainable exploitation of resources, particularly wildlife, was deemed achievable, assuming appropriate practices are put in place to regulate the hunting frequency and to target the most resilient species [48]. 

### Preliminary investigations

Before developing the EG, focus group discussions (FGDs) were conducted with hunters from 10 different communities participating in the Sustainable Wildlife Management (SWM)^1^ program (labelled A-J) in the Mulundu department. The objectives of these FGDs were to understand the context of hunting, the knowledge and perception of wildlife diseases by hunters and the feasibility of establishing an information reporting scheme enabling the notification of observed suspicions of zoonotic diseases to a health agency. After providing some background information on zoonoses and the risk of zoonoses transmission from wildlife, semi-structured interviews were conducted with a checklist of themes, including: (1) experience of hunters with unusual events in wildlife that could constitute suspect zoonotic diseases cases, (2) possible causes of these unusual events according to hunters, (3) communication channels that would allow the reporting of health information to a health agency, (4) barriers to the implementation of a surveillance system, and (5) perceived consequences of the control measure resulting from an alert on hunters' welfare. The objectives of the FGDs were clearly described to the hunters, notes were taken and the sessions were recorded to help the transcription. The interview guide and the investigation protocol are available in Supplementary Information S1 and S2.

### Game theoretical model

The expected utility theory stipulates that the individuals’ decisions are aimed at satisfying their preferences [49]. When a player is in the position of choosing among several options, he either has a single preferred option that is the most satisfactory for him – the one maximizing his utility—or is indifferent towards the outcome of two or more options with equivalent utility [43]. Non-cooperative games always have at least one equilibrium strategy called the Nash equilibrium [50]. The Nash equilibrium corresponds to a set of players' strategies where each player maximizes his utility given the strategies implemented by the other players [49, 50]. The Nash equilibrium needs to be distinguished from the societal optimum, which corresponds to a set of players’ strategies where the sum of the players’ utility—the societal welfare—is maximized. When decisions have externalities, some players may increase their individual utility by deviating from the societal optimum strategy, at the expense of other players' utility, as exemplified by the well-known prisoners' dilemma. This is true for most common instances of common or public goods management [51].

**Fig. 1:**
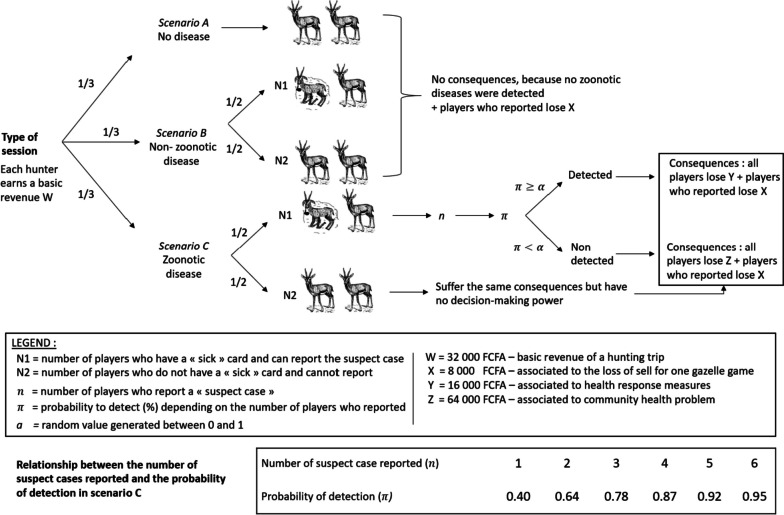
Scenario that can occur during the game sessions and consequences associated to the individual and collective decisions of the players; consequences are quantified in fictive monetary amounts (W; X; Z; Y); outputs related to the detection or non-detection of zoonotic diseases are dependent to the n, π and α values

We posit a system composed of a finite population of players. Players hunt animals of a given species on a daily basis for the purpose of home consumption and sale. The game is played in successive rounds corresponding to hunting trips during which players capture two animals. In a fraction of the rounds a disease event occurs causing the expression of clinical signs in a fraction of the hunted animals (Fig. 1). The disease event is either caused by a zoonotic disease or by a disease not transmissible to humans, but this information is not known by players. In rounds with disease events, a fraction of the players catches an animal displaying a set of clinical signs of a disease, latter referred to as a “suspect case” of zoonosis. These players must make a choice between two options: (1) keeping the animals for sale or consumption or (2) reporting the sick animal to a health agency. If choice (1) is made, the players obtain the totality of the revenue (W) of their hunting activity – i.e. the revenue from animal sales or the expenses saved in food purchase by consuming the captured animals. If choice (2) is made, they lose a fraction of their revenue (X) because the reported suspect animal cannot be used for sale or consumption. Each reported animal is tested for the presence of zoonotic diseases. Tests have a limited sensitivity meaning each test has a given probability of detecting the presence of a zoonotic pathogen comprised between 0 and 1 (*ρ*). Consequently, the probability of detection (π) increases with the number of reported suspect cases. Rounds with zoonotic disease events have two possible outcomes: (i) the disease is detected early because at least one test made on the animals reported by players is a success; or (ii) the disease is not detected, either because no players reported or because the test(s) made on reported suspect animal(s) failed to detect the disease. Outcome (i) causes every player to pay a cost (Y) because temporary control measures are implemented to limit the risk of human infection (e.g. ban on wild animal trade). Outcome (ii) causes all players to pay a higher cost (Z), as players suffer the consequences of the zoonotic disease outbreak, including human infections, with resulting loss of activity and medical expenses, and decreased sales of wild meat due to the control measures implemented or the reluctance of consumers to buy unsafe products. The game is static and rounds are independent, i.e. there is no correlation between probabilities of zoonotic or non-zoonotic disease events, and between the distribution of players who capture suspect animals in consecutive rounds.

In the used game settings, the probability of disease detection by a test is set to $\rho =0.4$. The probability to detect the zoonosis is: $\pi = 1 - {(1-\rho ) }^{n}$ with $n$ the number of players who report a suspect case (Fig. 1). The fraction $\gamma$ of disease events of zoonotic nature is set to $0.5$ (i.e. an equal occurrence of disease of zoonotic and non-zoonotic nature). The general expression of the utility of an individual player capturing a suspect animal in a given round is:

$${U}_{D,n}=W-{C}_{i}\left(D\right)-{C}_{c}(n, D)$$

With $W$ the basic revenue earned from hunting wild animals in a round, ${C}_{i}$ the individual cost incurred by the player which is a function of each reporting decision ($D$), and ${C}_{c}$ the collective cost incurred by all players in case of zoonosis, which is dependent on the number of other players reporting a suspect case ($n$) and the individual players’ decision ($D$). The expected revenue per round in case of reporting ($D=1$) and no reporting ($D=0$) are respectively:

$${U}_{1,n}=W-X-\gamma \left(\left(1-{\left(1-\rho \right)}^{n+1}\right)Y+{\left(1-\rho \right)}^{n+1}Z\right)$$

$${U}_{0,n}=W-\gamma \left(\left(1-{\left(1-\rho \right)}^{n}\right)Y+{\left(1-\rho \right)}^{n}Z\right)$$

And the difference between the anticipated revenue resulting from $D=1$ and $D=0$ is:

$${\Delta U}_{n}=\gamma \rho \left(Z-Y\right){\left(1-\rho \right)}^{n}-X$$

${\Delta U}_{n}$ is strictly decreasing in $n$, meaning that participation in the surveillance system is a strategic substitute. If more players report suspects cases for testing, the probability that the health agency fails to detect a zoonosis when it is present decreases. However, the difference in probability of failure is also decreasing in the number of suspect cases reports, so the marginal benefit of additional reporting is decreasing in the number of players reporting suspect cases. Based on this mathematical expression and the game parameters described (Fig. 1), it is possible to identify the unique Nash equilibrium and the societal optimum number of players reporting a suspect case in rounds with disease event (the full demonstration is in Supplementary Information S3). 

### Experimental game: practical implementation

One pilot session was played within the investigation team in order to test the game, adjust the rules and train the investigators in facilitating the game. One additional pilot session was implemented with a group of hunters in one of the communities. Then game sessions were implemented in 9 other communities with one group of hunters per community and one game session played per group. Two investigators facilitated the implementation of the game. One of the facilitators introduced the study and its objectives, explained the different steps of the game, re-explained whenever necessary, and reported to the participants the results obtained during each round. The other facilitator distributed and collected the game cards, reported the decision of players in an excel program, computed the outcome of each game round and the final scores of players.

### Characterisation of hunters

Before starting the game, personal badges were distributed to players, so that each player had one personal identification number (ID) that was displayed and visible to every other participant. An individual "hunter characteristics" questionnaire written in French language was filled by each player (with the help of the facilitator if needed). This questionnaire was designed to collect baseline information on the players’ involvement in hunting: (1) number of years of practice of hunting, (2) number of monthly catches, (3) sales volume, (4) hunted species, (5) economic activities outside of hunting. This questionnaire (Supplementary Information S4) was anonymous and participants only had to inform their attributed personal ID when filling the questionnaires.

### Game implementation

The rules were explained to the players. The game was played in 21 rounds, each round corresponding to a hunting trip. Duikers were chosen as the game animal species. The rounds were independent from each other. At the beginning of each round, the players received fictive monetary amounts of ( +) 32 000 FCFA (“Franc de la Communauté Financière Africaine”, the currency used in Gabon) corresponding to the revenue of a standard hunting trip (W). Players were given two cards per round representing duikers (suspect cases were distributed randomly and their repartition among players were determined in advance). A game board displaying a matrix with numbered individual cells corresponding to participants’ ID was used to ease the process of distribution and collection of game cards: at the beginning of each round the facilitators placed pairs of cards destined to each player on their corresponding cells, face down. At the start of each round, players collected the 2 cards placed in their dedicated cell.

Each individual player could receive two sets of cards: (1) two cards displaying healthy duikers, in which case no particular decision was needed and players had to return one of two healthy duiker cards; (2) one card displaying a healthy duiker and another displaying a sick duiker with clinical signs corresponding to a suspect case of zoonosis. The latter situation meant the player collected a suspect case during the hunting trip and had to decide whether to report it or not to the health agency. The player anonymously signalled his decision to the facilitators by returning either the healthy or the sick duiker card to the facilitators if the choice was to conceal or to report the suspect case respectively. The identity of players with suspect cases and their decision was unknown to other players. Each player placed the card they decided to return on to their dedicated boxes on the game board, face down.

In each round, three scenarios could occur with an equal probability (1/3) of occurrence: (A) no suspect case at all for all players (7 out of 21 rounds); (B) half of the players with a non-zoonotic suspect case (7 out of 21 rounds); (C) half of the players with a zoonotic suspect case (7 out of 21 rounds) (Fig. 1). The probability of occurrence of the three scenarios was set to be illustrative rather than realistic. Based on the results of the preliminary investigation, we knew the true frequency of encounter of sick animals was in reality smaller, but applying a realistic frequency of rounds with no decision making would have compelled us to substantially increase the duration of the game to gather the same amount of information. Additionally, no prior knowledge was available on the proportion of observable signs of sickness caused by zoonotic or non-zoonotic diseases.

There was no visible difference between “sick” duiker cards associated with a zoonosis or a non-zoonotic disease so that players could not know whether they were in scenario B or C when making their choice. For the players who decided to report, the penalty was (-) 8 000 FCFA (X). In rounds with zoonotic event (C), the collective cost incurred per player was (-) 16 000 FCFA (Y) and (-) 64 000 FCFA (Z) if the health agency succeeded or failed to detect the zoonotic disease respectively (Fig. 1). The probability of detection (π) depended on the number of reporting players (*n*) and the sensitivity of the test ($\rho$). A random value (α) was generated from a uniform probability distribution ranging from 0 to 1 for each round with scenario (C): if π < α the health agency failed to detect the zoonosis while if π ≥ α the health agency succeeded to early detect the zoonosis (Fig. 1). Rounds with scenarios A, B and C were randomly ordered and suspect case cards were randomly allocated to half of the players in each round with scenarios B and C. The ordering of scenarios and allocation of suspect cases cards per session were established in advance and differed across communities.

Three training rounds with each of the 3 scenarios were performed successively before the beginning of the game to make sure the participants correctly understood the rules.

At the end of each round, net results were calculated for all players. The facilitator orally reported to participants what had happened during the round: how many suspect reports were received, whether a zoonosis was present or not, whether it was detected early or not, and the resulting score for players having reported and not reported a suspect case. Then the next round was played.

### Debriefing of the game

Individual scores – the sum of the net results made by each participant across all the rounds—were announced at the end of the game. Explanations of the scores allowed the participants to better understand the collective interest of the players to report: the higher the number of reported cases, the higher the average score of the players in the community. In case of total absence of reports, the predicted average score per player is ( +) 512 000 FCFA over the entire game. In contrast, when all suspect cases are reported, the expected average score per player is ( +) 792 000 FCFA. A debriefing was conducted to collect the participants' perspectives and to better understand what type of behaviour they were ready to adopt towards the surveillance system. The participant’s feedback was recorded through note taking.

### Data analysis

#### Descriptive analysis

Individual decisions were collected in an Excel file. The numbers of reports per round with a disease event (scenario B or C) were compared to the theoretical Nash equilibrium—in this case 1.36 (between 1 and 2 players reporting suspect cases in practice)—and to the theoretical societal optimum, which depends on the number of players per community (Table 1). The societal optimum is equal to or higher than the number of players who have a "suspect case" card except when the number of players is higher than 12. Therefore, for the communities 12 players or less, the societal optimum actually corresponded to a situation of pure strategy where each player collecting suspect cases report it to the health agency.

**Table 1:** Relationship between societal optimum and number of players in the theoretical setting of the game. Number of suspect case card depending on the number of players is also presented (1/2 of the players) to understand the relationship between the number of players and the societal optimum

Numbers of players per community	5	6	7	8	9	10	11	12	13
Number of players with a suspect case card	2–3	3	3–4	4	4–5	5	5–6	6	6–7
Societal optimum	3.99	4.34	4.65	4.91	5.14	5.34	5.53	5.70	5.86

The average proportion of players with suspect cases who report was calculated for each community in the first 7 rounds and the last 7 rounds with a disease event (2/3 of 21 = 14 rounds with a disease event) in order to compare communities’ inclination to report and the evolution of their reporting attitude throughout the game.

### Statistical analysis

Multivariable logistic regressions were performed to assess the dependence of the choices made by players on (1) their hunter profile established from the “hunter characteristics” pre-game questionnaire and (2) events occurring during the game session. Two separate models were fitted:

- Model 1 (“player”): effect of the players’ characteristics on the proportion of suspect cases reported

The unit of observation was the player. A mixed-effect beta-binomial logistic model was implemented, the dependent variable being the number of suspect cases reported by the player over the game session, bounded by the number of rounds the player collected a suspect case, and the Gaussian random effect being the community. The independent variables were the player’s characteristics: (a) number of years of practice of hunting; (b) number of monthly catches; (c) sales volume (“a lot” i.e. collected animals more frequently sold than consumed; “few” i.e. collected animals less frequently sold than consumed; “none” i.e. no sale); (d) types of hunted species (duikers; porcupines; red-river hogs; monkeys; other); (e) number of activities associated with hunting ( “hunter”, “game porters”, “trapper” or “rifle owner”); (f) practice of an agricultural activity besides hunting (Yes; No); (g) practice of a fishing activity besides hunting (Yes; No). The three last variables were obtained from responses to the question on the economic activities of the player in the questionnaire (Supplementary Information S4).

- Model 2 (“choice”): effect of the players’ characteristics and game rounds on the choice made by players

The unit of observation was the binary choice (report or no report) made by players. A mixed-effect binomial logistic model was implemented, the binary dependent variable being the choices. The Gaussian random effect was the player and several additional random effects were tested, namely the player’s community and the community-round—i.e. the effect of a particular round in a particular community. The independent variables were (1) the characteristics of the players used in the model 1 and, in addition, (2) the characteristics of the rounds, including: (a) the number of the round (from 1 to 21); (b) the scenario of the previous round (scenario A; scenario B; scenario C with early detection; scenario C with late detection); (c) the total number of previous rounds with scenario A; scenario B; scenario C with early detection; scenario C with late detection; (d) the total number of reports in all the previous rounds and; (e) the number of reports in the last round with suspect cases (scenario B or scenario C).

In the two analyses, the full models (with all the random effects and independent variables) where implemented first. Then a stepwise *backward* and *forward* elimination of independent variables was performed, aiming at minimizing the model Akaike information criterion (AIC). In the model 2, the additional random effects were selected for inclusion in the final model on the basis of the comparison of the AIC of models incorporating or not incorporating these effects. The quality of the final models was tested with a chi-square goodness of fit test.

### Computing material

Microsoft Excel (Office 2016) was used for programming the game sessions, collecting the players’ choices and computing the game outcomes. R software [52] was used for the statistical analyses.

## Results

### Preliminary investigations

The full qualitative results from the preliminary study are presented in the report of Pouliquen [53]. Briefly, the results showed hunters occasionally observe unusual signs in wild animals that can be related to suspicions of diseases, including (i) mortality, (ii) weight loss and (iii) difficulty to walk. When it occurs, they commonly eat or sell the game but they can also leave it. The decision is sometimes taken after discussing with other members of the family. When enquired about their perception of a hypothetical health surveillance system, participants foresaw positive effects of reporting suspect cases to a health agency in terms of human health protection, but also several negatives effects associated with the possibility of disease announcement and resulting restrictions: (i) a reduction in wild meat consumption, (ii) economic losses due to lower sales of game products, and (iii) loss of dietary sources due to decreased hunting activities. Moreover, the hunters were reluctant to liaise with a public agency because of the informal nature of their activity. Indeed, most hunters had no legal licence authorizing hunting and gun ownership.

### Participants and game duration

Eighty-eight players participated in the game sessions (excluding the pilot session). Sessions gathered 10 players on average [min 5; max 13] (Table 3). Having an active hunting activity was the unique condition to participate in the games. In one community, porters—people who help the hunter transport the game—also participated, because of the low number of hunters present in the community. Opinion leaders such as village heads and presidents of community-based hunting associations were also invited to participate even though they were no longer hunting. All participants were men with the exception of one community, where a woman gradually played on behalf of an elderly hunter. Game sessions lasted approximately 3 h from the explanations of the rules to the debriefing.

### Descriptive statistics

#### Players profile

Players had on average a long experience in hunting, captured 9 animals per month – mostly duikers and porcupines –, and tended to use their captured game for home consumption rather than for commercial purpose. However, these characteristics varied substantially across players (Table 2). A bit more than half of participants practiced either agriculture or fishing or both activities besides hunting.

**Table 2:** Descriptive statistics of the characteristics of the players related to the questionnaires filed before the game

	**Mean**	**Standard deviation (sd.)**	**Minimum**	**Maximum**
**Duration of hunting activity (years)**	22	± 16	1	61
**Number of animals captured in a month**	9	± 10	0	50
**Quantity of game animals sold**^**a**^** (proportion of participants %)**	A lot = 25%	Few = 60%	None = 15%	
**Other activities besides hunting (number of participants *n*)**	Agriculture *n* = 37		Fishing *n* = 25	
**Hunted species (number of participants *n*)**	Duikers *n* = 68	Porcupines *n* = 50	Red-river hogs *n* = 21	Monkeys *n* = 17

^a^ : "a lot” i.e. collected animals more frequently sold than consumed; “few” i.e. collected animals less frequently sold than consumed; “none” i.e. no sale

#### Game sessions

41% of suspect cases were reported across all game sessions. The proportions of players who never reported, always reported, and reported only in a fraction of the game rounds where they collected a suspect case was 26%, 14% and 60% respectively.

**Fig. 2:**
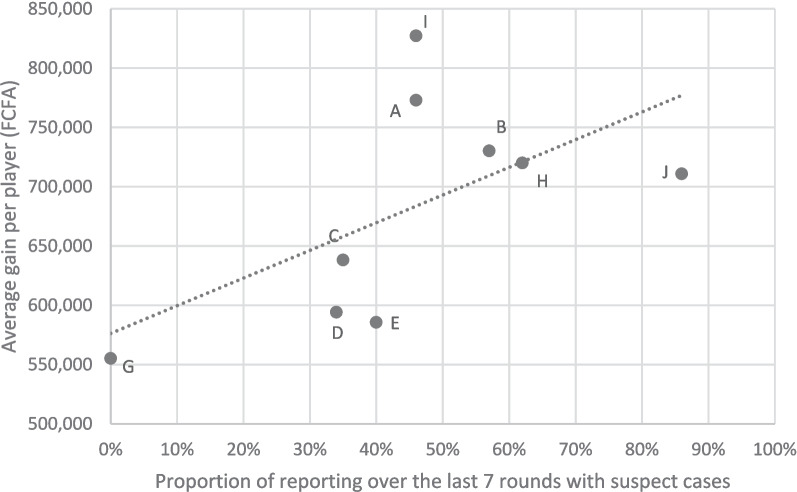
Graphical plot of the relationship between the proportion (%) of reported suspect cases over the last 7 rounds of the game sessions and the average score (FCFA) per player in the corresponding community. Communities are identified with their correspond corresponding community. Communities are identified with their corresponding letters. The dotted line represents the linear regression of the average gain on the average proportion of reported suspect cases

The average number of suspect cases reported per round with disease event was suboptimal from a social standpoint – i.e. below the societal optimum – in every communities (Table 3) but above the theoretical Nash equilibrium in every community except two (communities D and G). On average, the proportion of reporting players increased by 10% (standard deviation: 15%) between the first 7 and last 7 rounds with disease events. The average score per player was positively correlated with the proportion of suspect cases reported in the last 7 rounds at the community level (Fig. 2, Pearson correlation coefficient = 0.59). Communities D and C were the closest to Nash equilibrium and were also among those with the lowest proportions of reporting players (30% and 38% over the last 7 rounds with diseases events respectively) except community G where players never reported in the last 7 rounds with suspect cases. Communities A, H and J were the closest to their respective societal optimum and had the highest average scores.

**Table 3:** Analysis of game sessions based on community player average decision to report or not (average number of reports per round; proportions of reports of suspect cases first 7 and last 7 rounds with suspect cases; average win per player)

**Community**	**Number of players**	**Value of the societal optimum**	**Average number of reporting per round during disease events**	**Proportion of reports (first 7 rounds with disease event)** (a)	**Proportion of reports (last 7 rounds with disease event)** (b)	**Evolution of the proportion of reporting during the game** (b)-(a)	**Average win per player at the end of the game (FCFA)**
**A**	13	5.86	3.14	50%	47%	(-) 3%	772 923
**H**	12	5.70	3.42	52%	62%	( +) 10%	720 000
**B**	11	5.53	2.14	21%	55%	( +) 34%	730 181
**D**	11	5.53	1.35	18%	30%	( +) 12%	594 181
**E**	10	5.34	2.00	40%	40%	0%	585 600
**I**	10	5.34	1.85	29%	46%	( +) 17%	827 200
**C**	9	5.14	1.42	28%	38%	( +) 10%	638 222
**J**	7	4.65	2.57	65%	88%	( +) 23%	710 857
**G**	5	3.99	0.21	17%	0%	(-) 17%	555 200
MEAN				**36%** **(sd. 17%)**	**45%** **(sd. 24%)**	**( +) 10%** **(sd. 15%)**

### Statistical analysis

Three players were removed from the dataset because of errors in the distribution of their cards during the game session. Five additional players were removed because of missing data in their hunters’ characteristics questionnaire responses. The hunted species variable was not included in the statistical models because of the high number of missing or unclear responses to this question. 80 players were kept in the analysis.

**Fig. 3:**
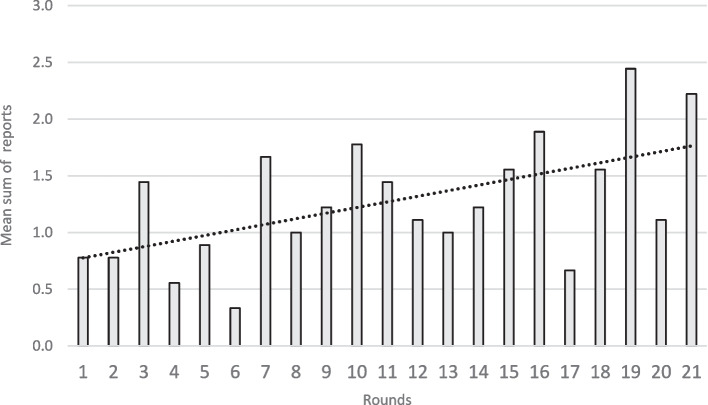
Evolution of the mean number of reported suspect cases during game sessions (all communities combined). Round numbers from 1 to 21 are displayed on the X axis. The bar plot shows the mean number of reports made by players over the 9 communities. The dotted line represents the linear regression of the mean sum of reports over the round number

The random effect (community) and only one fixed term were kept in model 1 (“player”). Only one random effect (player) and three fixed term effects were kept in model 2 (“choice”). According to the two fitted multivariable logistic models (Table 4), players who had an agricultural activity were significantly more likely to report suspect cases than other players. The outcome of model 2 showed that false alarms -suspect cases without zoonotic disease—led to fewer reports in the next round and that the frequency of reporting of suspect cases increased throughout the sessions (Fig. 3).

**Table 4:** Results of the two best multivariate logistic regression based on the lowest AIC (Model 1: effect of the players’ characteristics on the proportion of suspect cases reported; Model 2: effect of the players’ characteristics and game rounds on the choice made by players). A chi-square goodness of fit test confirms the validity of the final model

	**Model 1 (model « player»)**			**Model 2 (model « choice»)**		
	***OR***	***95% confidence interval***	***p-value***	***OR***	***95% confidence interval***	***p-value***
**Practice of agriculture**	2.05	[1.09;3.88]	< 0.03	4.13	[1.14;14.9]	< 0.04
**Rank of the game round (incremental OR)**	-	-	-	1.11	[1.06;1.17]	< 0.01
**Non-zoonotic disease in the previous round**	-	-	-	0.46	[0.26;0.80]	< 0.01
**Chi-square goodness of fit test**	-	-	< 0.01	-	-	< 0.01

## Discussion

To the best of our knowledge this is the first application of an EG to the topic of zoonotic diseases surveillance. While choice experiments were conducted to evaluate the factors affecting farmers’ decision to report suspect cases of livestock diseases [35, 36], these experiments were individual and did not address the collective dimension of surveillance participation and the strategic response of surveillance actors to the groups’ behaviour and to surveillance outcomes. The study is also one of the first to investigate the socioeconomic constraints to the implementation of a surveillance system in the context of wild meat exploitation. The recreational context created by the game encouraged the expression of the constraints that influence the hunter's choices in real situations. This context, added to the anonymity of the choices made by participants, limited the risk of desirability bias [54], as suggested by the low proportion of participants who always reported suspect cases. The results will be used to inform the design and implementation of a real community-based zoonotic diseases surveillance system in the same study site where the experiment was conducted.

This study was inspired by previous game theory and experimental studies on peoples’ adoption of health services [44–47, 55]. It was, however, implemented in a particular context, with hunters being unaware of health surveillance prior to the study. For this reason, we conducted a series of semi-structured FGDs with hunters before developing and testing the game. FGD results showed that hunters and their families might eat animals found sick or dead without any apparent reason, partly because they had limited or no perception of the zoonotic risk. This is consistent with the findings of a survey in Cameroun [56].

No real rewards were used in implementing the EG, i.e. the participants’ scores were not converted into real cash money or in-kind rewards. All participants were simply given refreshments at the end of the game sessions. There were two reasons for not using real incentives. First, the study was part of a program that conducts a wider range of activities with the same communities. Using real incentive in this study could have adversely impacted the project by creating expectations of rewards for participating in other activities. Second, the message delivered to communities might have been altered if the players had received real rewards, since the “free-riders”, who reported fewer suspect cases than others, would have received a higher financial gain than the other players. Our EG was conducted with all participants gathered in the same room with the freedom to interact, therefore communication between participants occurred during game sessions and some participants orally revealed their choices to other players or openly asked other players to report their suspect cases. A strict physical separation of participants would have been difficult to implement in practice and would have been negatively perceived by participants. Additionally, allowing interactions created a collective emulation that maintained a constant involvement of participants in the game [40] and allowed an exchange of opinions among participants on the benefits and disadvantages of participating in a surveillance system. These methodological aspects might affect the external validity of our results, as participants may have behaved in a less prosocial way (i.e. reported fewer suspect cases) if reporting would have meant conceding a true loss of reward. Additionally, the lack of perfect anonymity of the choices made by participants might have biased participants towards a more prosocial attitude than what would have been observed in a situation of strict confidentiality. In conducting an EG on the management of common water resources by farmers communities in India, Bartels et al. [38] found that using real incentives as opposed to hypothetical ones had no significant effect on the players’ behavior in game sessions—a result also obtained by Meinzen-Dick et al*.* [39] – but that participants behaved in a more cooperative way during game phases when communication and disclosure of players’ choices was allowed. Additionally, game sessions using real payments tended to have a slightly higher effect on subsequent management decisions in real life [38, 39].

We chose to use a common monetary metric to quantify the benefits of consuming or selling game, costs associated with policies restricting hunting activities in the face of an outbreak of zoonotic disease and costs associated with sickness, treatment, disability and hospitalization of community members resulting from an uncontrolled zoonotic disease outbreak. Those impacts are difficult to trade-off in decision making because of their differences of nature (financial, nutritional or medical). In attributing a monetary value to those benefits and costs, we allowed participants to rapidly and objectively evaluate the consequences of their decisions but, at the same time, we disregarded the heterogeneities in the preferences attributed to the nutritional value of wildlife consumption and to the avoidance of sickness among participants. If we had presented the outcome of the decisions of players in terms of impaired access to wild meat on one side and medical consequences of zoonotic diseases on the other side, instead of financial losses and gains, those heterogeneities of preferences would have certainly affected the decision making of participants.

In the game-theoretic model we developed—the theoretical basis of the EG—participation in zoonotic disease surveillance has positive externalities. The production of relevant information for the system is made at the expense of the players who report – and incur a cost – while it benefits the whole community [45, 48, 55]. The logical consequence is a suboptimal participation in surveillance i.e. a Nash equilibrium number of suspect cases reports below the societal optimum. This theoretical assumption was concordant with the results of the game sessions: even if the proportion of reporting increased throughout the game, it remained suboptimal from a social standpoint. Participation in surveillance is a strategic substitute, similar to vaccination decisions [46, 47]: the choice of reporting is expected to be preferred if the player anticipates an absence of participation of the other players. Indeed, the protection conferred to every single player by a single report outweigh the individual cost of reporting. However, as participation grows, additional suspect case reports have a decreasing effect on the probability of detection (π). In such a situation, players are tempted to act as “free riders”, i.e. not reporting, if they anticipate that at least one other player will report a suspect case. The results of the EG, however, does not support the existence of a true free-rider strategy among players. Indeed, the likelihood or reporting was not affected by the number of suspect cases reports received in previous rounds or the successful detection of zoonotic diseases in previous rounds. This result differs from experiments conducted on vaccination decisions [44–47, 55]. In the context of a game simulating surveillance, it was difficult for players to anticipate the future number of reports based on the outcome of previous rounds since rounds with disease events were alternated with rounds without disease events and the distribution of hunters collecting a suspect case varied from one round to another, consistent with real life situations. Strategic planning is certainly more difficult in the context of surveillance participation than in the context of vaccination, because of the high level of uncertainty people are facing on the prevalence and dangerousness of a health risk before it is reported, and therefore free-riding is probably less likely to happen in real life as well.

False alerts led to a significant decrease of reports in the next round. False alerts increase the perceived risk that the observed suspicious clinical signs are not due to zoonotic diseases and, therefore, lower the anticipated benefit of reporting. This strategic response of participants has significant implications as community-based surveillance systems always rely on suspect case definitions with limited specificity, which logically leads to false alerts. According to Wagner et al. [57] false alerts in disease surveillance systems are tolerable as long as their cost does not exceed the benefits derived from true disease detections. Our study results suggest that the risk of decreased participation resulting from excessive false alert rates must be accounted for in this assessment. Future community engagement activities must emphasize the usefulness of periodic false alerts to maintain a regular activity in surveillance systems and their functionality in the event of a true zoonotic disease outbreak.

Participants practicing agriculture were significantly more likely to report suspect cases than the others. Agriculture probably represents a substantial source of additional income and food for these hunters [16], making them less reliant on hunting for their livelihood and therefore less sensitive to the loss of revenue from hunting due to the report of suspect animals. This confirms the hypothesis that the willingness to participate in surveillance systems depends on the level of economic dependency of hunters towards wild meat [22, 58]. Understanding the socio-economic implications of decisions is critical to analyse the management of disease risks by households [13, 14, 16, 33]. For Gabonese communities who are dependent on hunting both for protein consumption and for covering household financial needs, surrendering a game carcass to a health agency may represent a high loss.

A sensitizing effect of the EG on participants is suggested by the progressive increase in the level of reporting of suspect cases throughout the game sessions. While playing, hunters progressively understood the benefits of reporting disease suspicions at the community level. However, we cannot exclude that gathering participants in the same room and allowing interactions between them gradually led to prosocial decisions resulting from peer pressure [39, 45, 59]. More in-depth studies would be needed to ascertain the effect of the EG on hunters’ behaviour in real life [38].

The developed EG could be adapted and extended to a larger panel of actors. Although, we considered each group of hunters represented their whole community, not all community residents played and all players were male. Additional participants could include women and children as well as foresters who also live and work at the human-wildlife interface. Zoonotic risks and their surveillance have implications for all community members and involvement in wildlife health monitoring should not be limited to hunters [22, 31].

## Conclusion

We demonstrate the relevance of EGs to improve our understanding of hunters’ decisions to participate in zoonotic diseases surveillance systems. Extending the implementation of the game to all potential actors of surveillance and replicating the methodology in other wild meat value chains in different contexts would provide useful information to support the development of community-based zoonotic risk management.

### Supplementary Information

**Additional file 1. **Presentation of objectives.

**Additional file 2. **Investigation protocol – Focus Group Discussion with hunters – Feasibility study to implement integrated community-based surveillance system of zoonosis in the wildlife supply chain in Mulundu, Gabon.

**Additional file 3. **Full demonstration : Nash equilibrium and societal optimum.

**Additional file 4. **Questionnaire – Surveillance game.

**Additional file 5. **Consent form : socio-economic survey on the feasibility and acceptability of a surveillance system for emerging zoonoses in wildlife in Gabon.

## Data Availability

The data collected in the pre-game questionnaires and during the game sessions were anonymised and is only accessible by the researchers directly involved in the study. Any disclosure of the data to a third party should be submitted for approval to the study participants (contact Alexis Delabouglise: alexis.delabouglise@cirad.fr).

## Abbreviations

AIC: Akaike Information Criterion
ASTRE: Animal Santé – Territoires – Risques—Ecosystèmes
CIRAD: Centre de coopération Internationale en Recherche Agronomique pour le Développement
CIRMF: Centre Interdisciplinaire de Recherches Médicales de Franceville
EG: Experimental Game
FCFA: Franc des Colonies Françaises d'Afrique
FGD: Focus Groups Discussion
ID: Identification Number
OR: Odds Ratio
SARS-CoV-2: Severe Acute Respiratory Syndrome Coronavirus 2
SWM: Sustainable Wildlife Management
UMR: Unités Mixtes de Recherche

## Footnotes

1 https://www.swm-programme.info/fr/homepage
