# Supplementary material for: An experimental game to assess hunter’s participation in zoonotic diseases surveillance
Source: BMC Public Health. 2024 Feb 1;24:342. doi: 10.1186/s12889-024-17696-7 (PMC10832086; doi:10.1186/s12889-024-17696-7)
Supplement: Supplementary file 1 — Additional file 1. Presentation of objectives. [file 12889_2024_17696_MOESM1_ESM.docx]

Presentation of objectives and consent forms for each actor

**Hunters:**

1. Focus on the main groups of hunted species, one after the other:

a. Antelopes and related species (including gazelles, antelopes, duikers)

b. Porcupine (Atherurus africanus)

c. Red-river hogs (Potamocherus porcus)

d. Monkey (mainly cercopithecines and mandrills)

e. Possibly add species according to hunters' suggestions

1. Notion of case: for all species

- Identify the problems hunters encounter with wildlife.

- Define the problems encountered, describe them and give examples of situations experienced.

- Identify the causes of the problems listed from the hunters' point of view.

- Determine whether there is mortality in the populations and the behavior adopted when encountering a carcass.

- Determine whether there is a seasonal pattern to the problems and assess the number of animals concerned.

Choose a species with a high mortality rate based on what is said, or suppose it.

1. Identify communication channels

- List a set of actors who could be warned if a surveillance system were set up, and note them on a post-it note (village chief, traditional practitioners, technical service, hunters' association, hunting guide, forest ranger, Community animal Health worker, other...).
- Make a proportional pile to elicit a discussion on their preferences (100 beans).
- Ask hunters if they can think of any other players with whom they do not communicate but could.

1. Present the purpose of the system at the end of the timeline, with the diagnosis that determines whether the disease affecting the animals is dangerous for humans or not, and if so, how dangerous the disease is and how it can be prevented.

- List a set of actions that can be carried out by these actors and note them on a post-it note (inform/warn, observe, protect, collect, etc.).

- Ask the hunters to place all these actors in chronological order (knowing that some actors may be at the same level). Also ask them to define the type of action being taken (is it information or control? etc.).

- Identify obstacles to communication with local authorities.

- Identify the obstacles and opportunities to such a communication system (means of communication, transport, trust, etc.).

- Which actors do you trust most in the process of setting up a response?

1. Identify the consequences of an alert for hunters (negative and positive).

During the activity, each time a new post-it note was placed, a picture was taken to see the evolution of the information chain during the discussions between the players.

A summary of the session is made with each group to review the objectives of the interview and the answers given:

- 1. Review the case definition.
  2. Summarize the actors with whom hunters can communicate and why (review motivations and obstacles).
  3. Show the information transmission timeline.
  4. Ask if there are any questions or comments.

After the session, participants were informed that the interviewers could come back if any details were missing or if any clarifications were needed following the session.

**Public decision-makers:**

1. *Identify zoonosis surveillance projects*

- Make a list of current and future zoonosis surveillance projects.

- Determine how information circulates and where this information is sent + Determine the type of information transmitted (monthly report, weekly "flash", report for immediate notification to the competent authorities).

- Identify how information is processed.

- Determine how monitoring systems are evaluated (operation: acceptability and fluidity / results: sensitivity and responsiveness).

- Identify the obstacles and opportunities for such a system (means of communication, transport, trust, etc.).

1. *Identify surveillance actors*

- Identify project initiators and participating actors (identify communication links between these actors).

- Identify sources of health information.

- Identify players who are not involved in the surveillance system, but who could potentially be part of it.

**Human health actors:**

*Laboratories (CIRMF, human health laboratory, etc.)*

- Determine how samples get to the laboratory and where the results go

- Determine what type of diagnostics useful for surveillance can be performed in the laboratory.

- Determine the diseases identified and the detection methods used (virology, serology, IgM, IgG).

- Identify where samples come from and who is involved in their collection.

- Determine how diagnostic information is circulated.

- Identify relationships with other surveillance actors.

*+ current projects*

*Hospitals, dispensaries + traditional medicine practitioners in villages*

1. *Determine the case notion*

- Determine whether diseases are transmitted by wild animals

- Determine whether zoonoses are identified and by which diagnosis.

1. *Identify channels for circulation of health information*

- If a diagnosis has been made, identify communication channels (means of communicating information, if any).

- Determine how the information collected by healthcare professionals is processed.

- Identify the obstacles and opportunities for such a communication system (means of communication, transport, trust, etc.).

1. *Identify surveillance actors*

- Identify relationships with other surveillance players.

**Animal health actors:**

*Veterinarian*

1. *Determine the case notion*

- Determine how suspected cases are identified and on which criteria.

1. *Identify channels for circulation of health information*

- Determine how information collected by animal health professionals is processed.

- Identify relationships with other surveillance actors.

- Identify sources of information on wildlife diseases.

- Know the consequences of an alert (positive and negative).

*Example: Positive = information and prevention*

*Negatives = loss of carcasses, reluctance of buyers*

1. *Identify zoonosis surveillance projects*

Make a list of current and future zoonosis surveillance projects.

- Determine how and where information circulates.

- Identify the obstacles and opportunities for such a communication system (means of communication, transport, trust, etc.).

**Environmental actors:**

1. *Identify zoonosis surveillance projects*

- Identify topics discussed with hunters.

- Make a list of current or future zoonosis surveillance projects.

- Determine how information is circulated and where.

1. *Identify surveillance actors*

- Identify relationships with other surveillance players.

- Identify sources of information on wildlife diseases.

- Identify the obstacles and opportunities of such a communication system (means of communication, transport, trust...).

- Know the consequences of an alert (positive and negative).

*Example: Positive = information and prevention*

*Negatives = loss of carcasses, reluctance of buyers*

1. *Identify channels for circulation of health information*

- Determine how health information collected by other actors is handled.

**Sage de village :**

1. *Determine the case notion*

- Determine the reasons why villagers or authorities contact the sage

- Determine how often hunters communicate with the sage.

- Determine whether wildlife is discussed with the sage, and for what reasons.

- Determine whether animal diseases or zoonoses are reported to the sage.

- Identify the nature of the sage's advice

- Identify how the sage uses the information he receives

1. *Identify surveillance actors:*

- Determine with whom the sage shares information

- Determine with whom the sage could potentially share information

- Determine what would be the obstacles and opportunities to disseminating information.

- From the sage's point of view, what are the consequences of sharing information?

o Positive consequences

o Negative consequences

1. *Identify communication channels:*

- Identify means of communication.

- Determine how health information collected by other actors is processed.

- How information is used by other actors
