# Supplementary material for: An experimental game to assess hunter’s participation in zoonotic diseases surveillance
Source: BMC Public Health. 2024 Feb 1;24:342. doi: 10.1186/s12889-024-17696-7 (PMC10832086; doi:10.1186/s12889-024-17696-7)
Supplement: Supplementary file 2 — Additional file 2. Investigation protocol – Focus Group Discussion with hunters – Feasibility study to implement integrated community-based surveillance system of zoonosis in the wildlife supply chain in Mulundu, Gabon. [file 12889_2024_17696_MOESM2_ESM.docx]

**Investigation protocol – Focus Group Discussion with hunters – Feasibility study to implement integrated community-based surveillance system of zoonosis in the wildlife supply chain in Mulundu, Gabon**

1. **Context – Bibliography – Justification**

Zoonotic diseases account for 75% of emerging and re-emerging diseases. In Gabon, wild meat is a significant source of protein and is preferred to meat from domestic livestock (pigs, chicken). Wild animals can constitute reservoirs and intermediary hosts for disease transmission to human.

Zoonotic disease surveillance is a matter of concern for the government and appears in the objectives of the National Health Development Plan 2017-2021. However, nothing has been clearly implemented to date.

To find out how and with which actors such a system could be set up, we need to make an inventory of current projects and carry out a feasibility study.

1. **Objectives and hypothesis**

*Objective :*

Determine the motivation of wildlife supply chain actors to participate in a zoonosis surveillance project (obstacles and drivers).

*Expected results:*

- Map the potential actors who could be involved in surveillance.
- Identify the obstacles and drivers to setting up a surveillance system.

The aim is to produce exploratory knowledge on the subject, even if numerous surveys on wild meat consumption and its zoonotic risks have already been carried out.

*Hypotheses:*

- There are a few programs aimed at inventorying pathogens and plans to set up zoonosis surveillance systems, but little resources available to achieve this. There is great interest in this type of project, but it is not a priority for the government.
- Potential surveillance players are interested in participatory surveillance programs because of their interest in human health, but are lacking resources (communication, transport, etc.).
- Hunters have little interest in participating in surveillance, as they are not highly aware of the zoonotic risks. Moreover, this type of participation can have a negative impact on their economic activity.

1. **Method**
2. **Study population**

Hunters from the 10 pilot villages located in the study areas where CIRAD activities are being implemented.

1. **Study area**

The 10 pilot villages are located in the Mulundu department, with Lastourville as its capital.

Selection criteria: villages with substantial hunting activity, a good partnership with the SWM program and a diversity of hunter types (Bantu and indigenous, subsistence and commercial). Ensure FGDs participants (Focus Group Discussion) as homogeneous as possible.

Plan a pilot FGD to test the FGD interview protocol.

1. **Data collection**
   1. **Participatory survey**

Qualitative method using semi-structured, individual or focus group interviews. The participatory approach is based on the knowledge and perception of local actors of the various issues at stake, in order to find solutions adapted to the context. This knowledge can be used to set up a reliable surveillance system.

- 1. **Survey collection**

Survey period from 01/03/22 to 27/05/22. Collection carried out by Aude Pouliquen and the project staff on site. Translation will be done if necessary. Recording of interviews and transcription before analysis.

- 1. **Ethical and regulatory aspects**

Consent form signed by participants authorizing note-taking and recordings. Approval from a CIFOR ethics committee.

1. **Data analysis**
   1. **Software used**

Word, Excel, R studio.

- 1. **Bias**

Several biases are identified prior to the surveys

*Representativeness and precision of the sample*: the number of participants to be interviewed cannot be determined in advance.

- Solution: to avoid bias, triangulation and saturation are required.

*Translation bias*: when translating, certain words are not necessarily used in the other language, and this can constitute a bias.

- Solution: Take as many notes as possible and record. If any of the answers are unclear or confusing, remove them for analysis.

*Social class bias:* people designated as the most influential or the most likely to take part in surveys on the same subject.

1. Hunters have already taken part in the SWM project, and are therefore familiar with the subject of sustainable wildlife management and collaboration with institutes.
2. People below the hierarchy may tend to talk less.

- Solutions :

(1) Don't ask questions that are too subjective, and refocus the discussion on the objectives of the survey. If the discussion moves away from the subject and focuses on issues not related to the objectives, redirect the discussion.

(2) Give each person the floor for each question; if a respondent is identified as having an opinion that could bring a new perspective, it is possible to conduct an individual interview with the this respondent, or conduct informal exchanges.

*Professional bias:* the interviewers' knowledge of the subject may lead to under- or over-interpretation of the facts being explained.

- Solution: stick to the facts and be as objective as possible.
