# Supplementary material for: An experimental game to assess hunter’s participation in zoonotic diseases surveillance
Source: BMC Public Health. 2024 Feb 1;24:342. doi: 10.1186/s12889-024-17696-7 (PMC10832086; doi:10.1186/s12889-024-17696-7)
Supplement: Supplementary file 3 — Additional file 3. Full demonstration : Nash equilibrium and societal optimum. [file 12889_2024_17696_MOESM3_ESM.docx]

The general expression of the utility of an individual player capturing a suspect animal in a given round is:

$$U_{D,n}=W-C_{i}\left( D \right)-C_{c}(n, D)$$

With $W$ the basic revenue earned from hunting wild animals in a round ($W=32000 FCFA$), $C_{i}$ the individual cost incurred by the player which is a function of his reporting decision ($D$), and $C_{c}$ the collective cost incurred by all players in case of zoonosis, which is dependent on the number of other players reporting a suspicion ($n$) and the individual reporting decision ($D$). The expected revenue per round in case of declaration ($D=1$) and no declaration ($D=0$) are respectively:

$$U_{1,n}=W-X-\gamma\left( \left( 1-\left( 1-\rho\right)^{n+1} \right)Y+\left( 1-\rho\right)^{n+1}Z \right)$$

$$U_{0,n}=W-\gamma\left( \left( 1-\left( 1-\rho\right)^{n} \right)Y+\left( 1-\rho\right)^{n}Z \right)$$

$X$ being the penalty incurred by hunters who declare ($X=8000 FCFA$), and $Y$ and $Z$ the penalties incurred by every player in case of a zoonotic disease detected early ($Y = 16000 FCFA$) or late ($Z=64000 FCFA$) respectively. $p_{Z}$ is the fraction of disease events of zoonotic nature ($\gamma=0.5$) and $\pi$ is the probability of detection of the zoonotic diseases following the report of a single suspicion case due to a zoonotic disease ($\rho=0.4$).

We can study separately three types of Nash equilibrium: a « pure » strategic equilibrium in which no player report (1) or every players report (2), and a “mixed” strategic equilibrium where a non-null fraction of players report (3).

Strategy 1 is a Nash equilibrium if and only if $X>\gamma\rho\left( Z-Y \right)$ i.e. if the individual cost of penalty associated with reporting is higher than the benefit of early detection of a zoonotic disease in case nobody reports suspicions. Given the parameters used in the experimental game, this inequality is wrong.

Strategy 2 is a Nash equilibrium if and only if $X<\gamma\rho{\left( Z-Y \right)\left( 1-\rho\right)}^{N-1}$, with $N$ the total number of players with a suspect animal and faced with the possibility of reporting. This inequality is true if $N\geq2$ and wrong if $N>1$.

Consequently strategy 3 is a Nash equilibrium when $N\geq2$ and the number of players $\bar{n}$ who report their suspicion at equilibrium is to the value of $n$ that satisfies the following equality:

$$X=\gamma\rho\left( 1-\rho\right)^{n-1}\left( Z-Y \right)$$

In other word, the individual cost of the penalty associated with reporting $X$ is equal to the benefit of early zoonotic disease detection when $n-1$ players report. The Nash equilibrium $\bar{n}$ is :

$$\bar{n}= \frac{1}{log\left( \frac{1}{1-\rho} \right)}log\left( \frac{\gamma\rho\left( Z-Y \right)}{X} \right)+1$$

In this case it corresponds to $\bar{n}= 1.36$, the average number of reporting players must be between 1 and 2.

The societal optimum is the value of $n$ which maximizes the sum of the revenues of all players – the social welfare. From a societal standpoint, it is beneficial that more many players report as long as :

$$X<-N_{T}\frac{dC_{C}}{dn}$$

With $N_{T}$ the total number of players. In other words, it is beneficial for the community that one additional player reports his suspicion if and only if his reporting reduces the collective cost of zoonotic diseases for the community by a higher amount than the individual cost $X$ he must incur for reporting. And:

$$-N_{T}\frac{dC_{C}}{dn}={N_{T}p}_{Z}\pi\left( Z-Y \right)log\left( \frac{1}{1-\pi} \right)\left( 1-\pi\right)^{n}$$

The marginal benefit of suspicion reporting for the community is a positive and strictly decreasing function of $n$. There is a single value of $n$ maximizing social welfare, dependent on the number of players $N_{T}$:

$$\hat{n}= \frac{1}{log\left( \frac{1}{1-\rho} \right)}log\left( \frac{N_{T}\gamma\left( Z-Y \right)log\left( \frac{1}{1-\rho} \right)}{X} \right)$$
