# Supplementary material for: An experimental game to assess hunter’s participation in zoonotic diseases surveillance
Source: BMC Public Health. 2024 Feb 1;24:342. doi: 10.1186/s12889-024-17696-7 (PMC10832086; doi:10.1186/s12889-024-17696-7)
Supplement: Supplementary file 4 — Additional file 4. Questionnaire – Surveillance game. [file 12889_2024_17696_MOESM4_ESM.docx]

**Questionnaire – Surveillance game**

Hello. Thank you for taking part in this surveillance game.

Before we start, we would like to gather some information about your hunting activity.

1. Participant number: _______________________________

2. Number of years of hunting activity: ________________________________

3. Sales:

- Nothing
- A bit
- A lot

4. What are your professional activities?

- Hunting
- Agriculture
- Fisher
- Village chief
- Group of village chief
- Notable village
- Rifle owner
- Other (specify) : _____________________________________________

5. Animal(s) mean quantity hunted in one month?

- (0)
- I (1)
- II (2)
- IIIII (5)
- IIIIIIIIII (10)
- IIIIIIIIIIIIIII (15)
- IIIIIIIIIIIIIIIIIIII (20)
- IIIIIIIIIIIIIIIIIIIIIIIIIIIIIIIII (30)
- IIIIIIIIIIIIIIIIIIIIIIIIIIIIIIIIIIIIIIII (40)
- IIIIIIIIIIIIIIIIIIIIIIIIIIIIIIIIIIIIIIIIIIIIIIIIII (50)
- IIIIIIIIIIIIIIIIIIIIIIIIIIIIIIIIIIIIIIIIIIIIIIIIIIIIIIIIIIIIIIIIIIIIIIIIIIIIIIIIIIIIIIIIIIIIIIIIIIII (100)


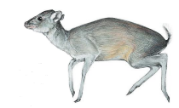
6. First specie hunted:

- Duikers


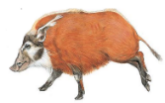


- Red-river hogs
-
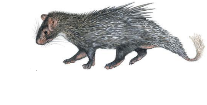

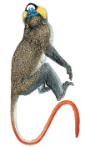
Porcupines
- Monkey
- Other


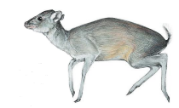
7. Second specie hunted :

- Duikers


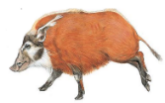


- Red-river hogs
-
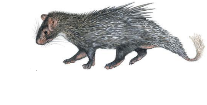

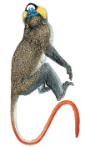
Porcupines
- Monkey
- Other
