# Supplementary material for: An experimental game to assess hunter’s participation in zoonotic diseases surveillance
Source: BMC Public Health. 2024 Feb 1;24:342. doi: 10.1186/s12889-024-17696-7 (PMC10832086; doi:10.1186/s12889-024-17696-7)
Supplement: Supplementary file 5 — Additional file 5. Consent form : socio-economic survey on the feasibility and acceptability of a surveillance system for emerging zoonoses in wildlife in Gabon. [file 12889_2024_17696_MOESM5_ESM.docx]

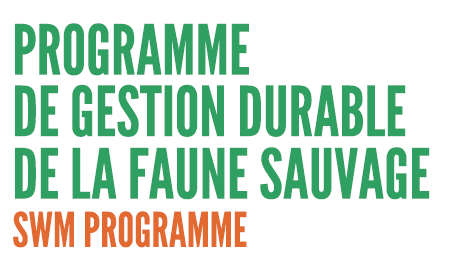

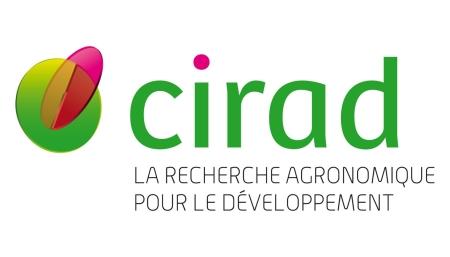


**Informed consent form**

**-**

**Socio-economic survey on the feasibility and acceptability of a surveillance system for emerging zoonoses in wildlife in Gabon**

**-**

**2022**

**Project team members:**

Gilles Boupana

Aude Pouliquen

**Purpose of the study:**

We invite you to take part in a research project based on an experimental game. Informed consent is required from participants.

Before you agree to take part in this game, please take the time to read the following information so that you know whether you want to participate or not.

This game is part of the SWM (Sustainable Wildlife Management) program, funded by the European Union, with the aim of better understanding current wildlife management and implementing sustainable management. Information is gathered by CIRAD (Centre de Coopération Internationale de Recherche Agronomique pour le Développement), a research organization based in Montpellier, France, in cooperation with Gabonese research organizations.

Some diseases present in animals, known as zoonoses, can be transmitted to humans.

In Gabon, wild meat is an important source of protein, and is preferred to meat from domestic farms (pork, chicken). Wild animals can be vectors for the transmission of diseases to humans through the handling and consumption of wild meat. The government has already set up warning systems for diseases that can be transmitted from animals to humans (Ebola, rabies). But for the time being, nothing has been done at wildlife level to target other suspected diseases.

As part of the health component of the SWM project, you are invited to take part in a zoonosis sensitization game.

During this activity, notes and photos will be taken.

**Data protection:**

Data will be collected by Gilles Boupana and Aude Pouliquen and shared with the CIRAD research team and other partners. We guarantee that the data collected for the study will be protected.

Use of the data will be restricted to the above study, and under no circumstances will it be given to private organizations or used for private purposes.

Access to survey data will be restricted to study researchers. The data will be kept confidential in a secure file.

The data analyzed will be anonymized for publication purposes: the names, locations and contacts of participants will not be mentioned. No link will be made between this consent document and the data, except for the project coordinators.

Data may be deleted in the future if you wish. The data will be kept only for the time needed to produce and publish the scientific results. It will then be destroyed. In accordance with Regulation (EU) 2016/679 of the European Parliament and of the Council of April 27, 2016 on the protection of individuals with regard to the processing of personal data and on the free movement of such data (General Data Protection Regulation) and Law No. 78-17 of January 6, 1978 on information technology, you have the right to access, rectify, delete and port your personal data (if applicable), as well as the right to limit and object to their processing for legitimate reasons. You can exercise these rights by contacting the Data Protection Officer at the following address XXXXXXX . You also have the right to lodge a complaint with the CNIL (Commission Nationale de l'Informatique et des Libertés) at any time.

L The information collected as part of this game will be kept in the best conditions of security and confidentiality throughout the research period, until the end of 2023. At the end of this period, personal data will be archived, and will be completely anonymized in 2030 once the link with the informed consent forms has been destroyed.

| **Data** | **Shelf life** | **Access conditions** | **Justification** |
| --- | --- | --- | --- |
| Consent form | 8 years (2030) | CIRAD researchers and SWM program partners; publishers | Archived for audit purposes |
| Results | Unlimited | No restrictions | No personal data |

If you wish to enforce your rights or obtain information about yourself, please contact the following person: XXXXXXXXXXXXXXXXX.

If, after having contacted us, you consider that your rights with regard to information and your civil liberties have not been respected, you can file a complaint with the CNIL by post: Commission Nationale de l'Informatique et des Libertés 3 Place de Fontenoy - TSA 80715 - 75334 PARIS CEDEX 07 or online at <http://www.cnil.fr/>.

**Game interruption:**

The maximum duration of the game is three hours. Participation is voluntary, and once you have given your consent, you can always ask for a break during the game or interrupt it definitively at any time.

**Consent:**

In order to guarantee your right to privacy, we ask you to give your explicit consent:

1. I have read and understood the above information.
2. I have had time to think about my participation in this game and I am aware that my participation is voluntary.
3. I agree that photos of the game may be taken (unless the person indicates that he/she does not wish to appear in the photos) for use by program researchers.
4. I agree that all information gathered in the course of this activity may be used free of charge in scientific or popular publications, provided that it is anonymized.

I have taken note that I can cancel my consent at any time by contacting the project manager mentioned below.

XXXXXXXXXXXXXXXXXXXXXXXXXXXXXXXXXXXXXX

Name(s) of participant(s):

| **Name and surname of participant** | **Number of participant** | **Agree to appear in photos** |
| --- | --- | --- |
|  | 1 | Yes/No |
|  | 2 | Yes/No |
|  | 3 | Yes/No |
|  | 4 | Yes/No |
|  | 5 | Yes/No |
|  | 6 | Yes/No |
|  | 7 | Yes/No |
|  | 8 | Yes/No |
|  | 9 | Yes/No |
|  | 10 | Yes/No |
|  | 11 | Yes/No |
|  | 12 | Yes/No |
|  | 13 | Yes/No |
|  | 14 | Yes/No |
|  | 15 | Yes/No |
|  | 16 | Yes/No |
|  | 17 | Yes/No |
|  | 18 | Yes/No |
|  | 19 | Yes/No |
|  | 20 | Yes/No |

Date: Name of signatory/Signature:
